# Deep learning multi-organ segmentation for whole mouse cryo-images including a comparison of 2D and 3D deep networks

**DOI:** 10.1038/s41598-022-19037-3

**Published:** 2022-09-07

**Authors:** Yiqiao Liu, Madhusudhana Gargesha, Bryan Scott, Arthure Olivia Tchilibou Wane, David L. Wilson

**Affiliations:** 1 Department of Biomedical Engineering, Case Western Reserve University, 10900 Euclid Avenue, Cleveland, OH 44106 USA; 2 BioInVision Inc, Suite E 781 Beta Drive, Cleveland, OH 44143 USA; 3 Department of Radiology, Case Western Reserve University, 10900 Euclid Avenue, Cleveland, OH 44106 USA

**Keywords:** Experimental models of disease, Bioinformatics, Imaging, Software

## Abstract

Cryo-imaging provided 3D whole-mouse microscopic color anatomy and fluorescence images that enables biotechnology applications (e.g., stem cells and metastatic cancer). In this report, we compared three methods of organ segmentation: 2D U-Net with *2D-slices* and 3D U-Net with either *3D-whole-mouse or 3D-patches*. We evaluated the brain, thymus, lung, heart, liver, stomach, spleen, left and right kidney, and bladder. Training with 63 mice, *2D-slices* had the best performance, with median Dice scores of > 0.9 and median Hausdorff distances of < 1.2 mm in eightfold cross validation for all organs, except bladder, which is a problem organ due to variable filling and poor contrast. Results were comparable to those for a second analyst on the same data. Regression analyses were performed to fit learning curves, which showed that *2D-slices* can succeed with fewer samples. Review and editing of *2D-slices* segmentation results reduced human operator time from ~ 2-h to ~ 25-min, with reduced inter-observer variability. As demonstrations, we used organ segmentation to evaluate size changes in liver disease and to quantify the distribution of therapeutic mesenchymal stem cells in organs. With a 48-GB GPU, we determined that extra GPU RAM improved the performance of 3D deep learning because we could train at a higher resolution.

## Introduction

Cryo-imaging is a preclinical section and imaging technique that provides single-cell resolution (as good as 5 µm) and large fields of view (up to whole mouse or even whole rat) with 3D color anatomy and molecular fluorescence image volumes^1^. Cryo-imaging is utilized in a variety of biotechnology applications. For example, it has been used to quantify cancer imaging agents^2,3^, distribution of therapeutic cells^4,5^, and theranostics^6^. High-resolution cryo-imaging of a whole-mouse can produce as much as 120 GB of data, which makes manual analysis a daunting task. We previously created a method for automatic segmentation of fluorescent protein-labeled metastases^7^ and fluorescent-labeled stem cells^5^, which enables the quantification of cells labeled with dyes, quantum dots, and fluorescent proteins. Automatic organ segmentation is required to enable further analysis and quantification of organ distributions in such applications.

Deep learning-based organ segmentation has proven to be the state-of-the-art approach to many tasks. Multi-organ segmentations in humans are generally performed for organs and tissues of the abdominal region^8^, brain^9^, heart^10^, and pelvic region^11^. It is challenging because the organs of interest are of various sizes; smaller structures generally have worse performance. Aside from differences in organs of interest and imaging modalities, there are major differences in deep learning approaches, which include three aspects: dimensionality of input image, convolutional neural network (CNN) architecture, and post-processing. Input data can be 2D, 2.5D (multi-view 2D), and 3D images where images could be whole view or patches. Two-dimensional and 2.5D whole view images are input into 2D CNNs. Several studies have been conducted using 2D input^8,12^. However, it is widely believed that 3D input offers additional volumetric information that is important for the segmentation task. However, 3D CNNs use many more parameters than 2D CNNs, which poses a challenge for training (i.e., the optimization of parameters and memory constraints). Researchers employ 3D patch input^13^ because it reduces input size while maintaining high-resolution. But this method suffers from a restricted field of view, compared with a full 3D approach. Downsampled 3D whole volume input has previously been used^14^. CNN architectures for organ segmentation are generally based on fully connected one-stage or two-stage networks. A one-stage network, such as DenseVNet^15^, trains one CNN model for multi-organ segmentation. Thus, it is more time-efficient and memory-efficient than a two-stage network. Two-stage networks reduce background and enhance discriminative information for target organs. Roth et al.^13^ used a first stage output to restrict the input region of interest for a second stage. They showed that this cascaded approach gives beneficial results for challenging organs, such as the pancreas and small organs and vessels, with Dice score improvements of > 10%, compared with the one-stage results. Wang et al.^8^ used a first stage output to generate an attention map that was combined with the original image as input to the second stage. For the two stages, two different CNNs were used; parameters were optimized simultaneously using backpropagation. They compared their model with 3D U-Net^16^ and demonstrated Dice score improvements of at least 7% across all abdominal organs of interest. Qiu et al.^17,18^ utilized the first stage VNet to generate a low resolution segmentation of mouse embryo brain ventricle^19–21^ and body in 3D high frequency ultrasound images, followed by refinement using a second VNet in high resolution at the second stage. Isensee et al.^22^ hypothesized that a well-configured U-Net is still difficult to surpass. They did not apply new architectural variations but focused instead on using rules to automatically configure parameters, such as network topology (including whether to apply cascade learning or not), resampling factor, patch size, and mini-batch size, based on input data size, resolution, and available GPU memory. They claimed that full-resolution 3D U-Net is the best configuration overall, surpassing 2D U-Net and 3D U-Net cascade for analysis of 18 clinical in vivo imaging datasets. Common post-processing methods include connected components, level set^23^, and conditional random field^24^. The studies mentioned previously utilized clinical human images obtained using computed tomography^8,10,11,13,15,22–24^ (CT) or magnetic resonance imaging^9,10,12,14,22^. Our particular interest is in mouse imaging, where changes in organ location due to pose are more of an issue than with human images.

Recently, Schoppe et al.^25^ developed a 2D U-Net approach for multi-organ segmentation, named AI-based Mouse Organ Segmentation (AIMOS), for mouse CT scans. They segmented the brain, lung, heart, liver, kidneys, spleen, bladder, stomach, and intestine, with average Dice scores of 88% and 89% for native micro-CT scans and contrast-enhanced micro-CT scans, respectively. They further showed the applicability of AIMOS to light sheet microscopy scans of cleared mice. Although cryo-imaging has more informative contrast than these modalities, the results encouraged us to test 2D U-Net for our application.

We investigate the applicability of deep learning for segmenting mouse organs in block-face cryo-image volumes. There are no reports for such organ segmentation of block-face images, except for our very preliminary report^26^. AIMOS^25^ proved that 2D U-Net is good for organ segmentation in mouse CT images. However, 3D U-Net has been shown to be generally better than 2D U-Net for human organ segmentation (in nnU-Net)^22^. In this report, we create methods for segmenting cryo-image volumes. The organs of interest are the brain, thymus, lung, heart, liver, stomach, spleen, left and right kidney, and bladder. We applied deep stacked transformation^27^ for image augmentation, to improve the generalizability of deep learning models. In addition, we conducted some interesting sub-studies. First, we compared the segmentation performance of three methods: 2D U-Net with *2D-slices* and 3D U-Net with either *3D-whole-mouse* or *3D-patches* input data. There has been no previous head-to-head comparison as a function of the number of training samples. Second, we evaluated the number of training samples required for such methods, including regression analyses for learning curves^28,29^. Third, we tested for the best approach to improve segmentation, using a GPU with a large amount of RAM (48 GB).

## Multi-organ segmentation algorithm

### Preprocessing

Preprocessing steps include down sampling of full-resolution cryo-images, semi-automatic cropping of the downsampled volumes, and generation of deep learning data using input data from *2D-slices*, *3D-whole-mouse*, and *3D-patches*. Color cryo-images are downsampled from full-resolution by 8 × 8 × 4 in the x, y, and z directions, respectively, giving a resolution change from 10 × 10 × 40 µm to 80 × 80 × 160 µm. To semi-automatically crop the downsampled color cryo-images from the nose to the tail region, we render 1/5–4/5 of the 3D volume in the z dimension and determine a tight cropping range to minimize the input size. For *2D-slices* input, all 3D volumes are sliced into coronal slices, then resampled to the size of 768 × 256 voxels. The *3D-whole-mouse* data are generated by resampling the cropped volumes to the size of 384 × 128 × 64 voxels. The *3D-patches* data are generated by using a sliding window in the cropped 3D volumes, with a window size of 288 × 96 × 48 and an overlap of 72 × 24 × 12 between neighboring patches in the x, y, and z directions, respectively. The cropped volumes are padded with zeros on the border in order to retain uniform patch size. All inputs are rescaled to an intensity range of 0–1 by multiplying each color channel by a factor of 1/255. The original 3D mouse image data required 35 GB of storage. We effectively analyze color data for each mouse using 120 MB, 10 MB, and 140 MB for *2D-slices*, *3D-whole-mouse*, and *3D-patches* input data, respectively.

### CNN-based segmentation

**Figure 1:**
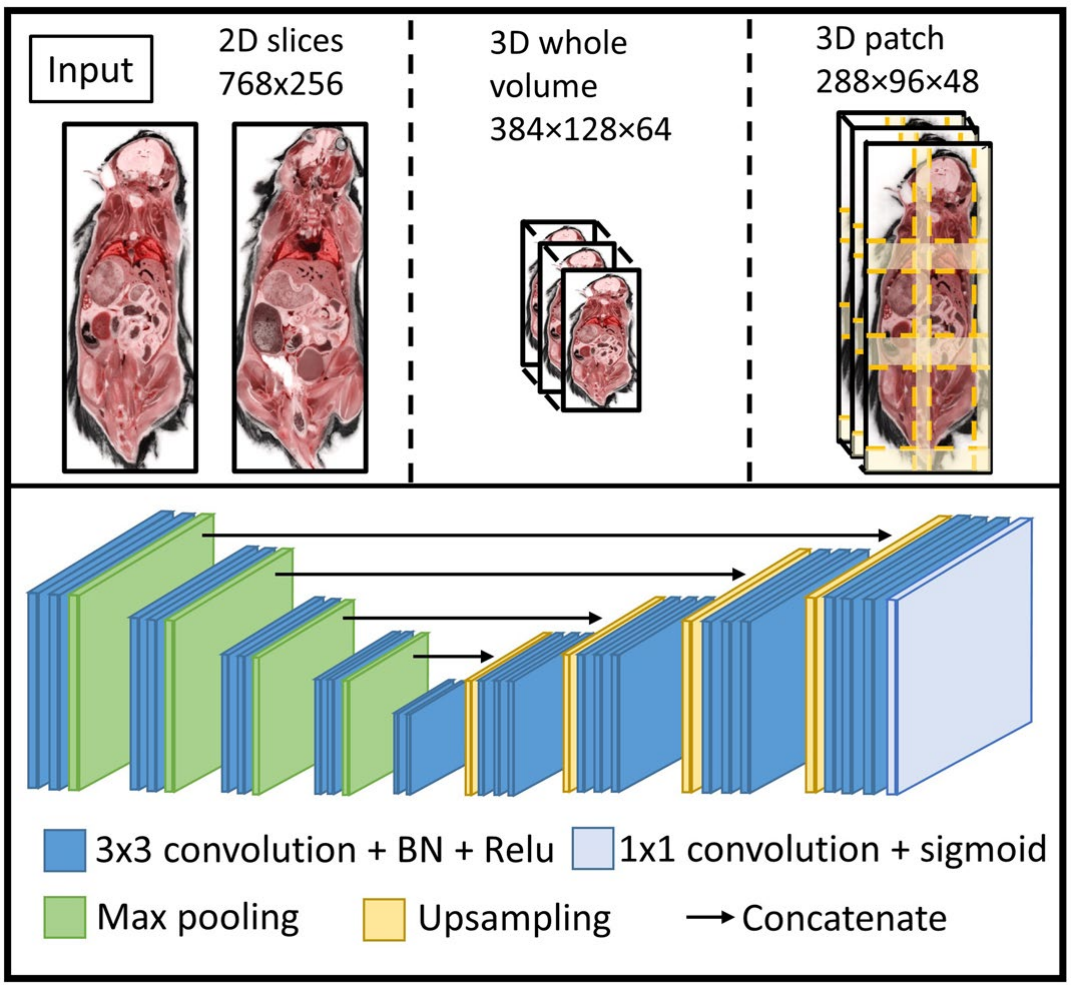
Overall workflow of organ segmentation. (Top) The input configurations for the three proposed methods. (Bottom) The network architecture for 2D U-Net. 3D U-Net has same architecture, except for the kernels being three dimensional.

The CNN has a U-Net-based backbone, with encoding and decoding paths connected by skip connections, as shown in Fig. 1. At each encoding level, there are two convolutions (kernel size, 3; padding, 1; stride, 1), each followed by batch normalization, a rectifying linear activation (Relu) function, and a max pooling layer. Each level has twice the number of kernels as the previous level; there are 16 kernels at the first level. Each decoding level consists of three convolutions, each followed by a Relu function. The first convolution receives input from the upsampled input of the previous level and from the corresponding skip connection in the encoding level. Each decoding level has the same number of kernels as the skip-connected encoding level. A final sigmoid function is added to generate probability map output for each organ. Dice score loss is used as a loss function to account for the different sizes of the target organs, as follows:

1 $$Dice\, loss=1-2\frac{\sum_{i=1}^{n}{R}_{i}\cdot {P}_{i}}{\sum_{i=1}^{n}{R}_{i}+{P}_{i}},$$

where *R*_*i*_ is the ground truth label for the ith organ, *P*_*i*_ is the predicted probability for the ith organ, and *n* is the total number of organs of interest, plus the background (n = 11, in our case).

Deep stacked image augmentation of image quality and appearance were applied, along with spatial transformation^27^. Image augmentation improves the generalizability of models and their segmentation performance on an unseen dataset. We use augmentations to simulate the anticipated variability of datasets. A probability of 0.5 is associated with each type of augmentation.

For image quality augmentations, we adjust blurring, sharpness, and noise. Gaussian filtering was applied for blurring, with σ ranging 0.1–0.5 voxels. Unsharp masking was utilized for the sharpening operation, with α ranging 2–10, as follows:

2 $${I}_{sharpened}={I}_{blurred}+\left({I}_{blurred}- {I}_{filteredblurred}\right)\times \alpha ,$$

where *I*_*blurred*_ and *I*_*filteredblurred*_ are blurred images obtained by applying Gaussian filtering on images I and I_blurred_, respectively. Gaussian noise, with standard deviation ranging between 0 and 0.01, is applied to data, based upon a normalized intensity range of 0–1. Image appearance, brightness, and contrast are adjusted separately for each color channel. The brightness shift range is 0.8–1.2. Contrast is computed using Eq. (3), as follows, where factor β ranges 0.6–1.05, with the higher bound set at 1.05 to avoid saturation in some bright tissue regions, such as the brain.

3 $${I}_{contrast}=\left[I-mean\left(I\right)\right]\times \beta +mean\left(I\right),$$

where *mean(I)* is the mean intensity of one color channel of the image. Spatial transformations are applied, including rotation and scaling. Two-dimensional spatial transformations are applied to *2D-slices* input, while 3D spatial transformations are applied to *3D-patches* and *3D-whole-mouse* inputs. We apply stacked augmentations so that, for any sample, there is a potential for seven augmentation operations in the following order: rotation, scaling, brightness, contrast, blurring, sharpness, and add noise. As a result, there is a potential that input data could be rotated, not scaled, brightened, contrast-enhanced, blurred, not sharpened, and with no noise added.

### Post-processing

With 10 organs of interest to segment, the deep learning networks generate predictions for 11 classes (10 organs + background). The post-processing steps for the predicted probability maps are: (1) create 3D whole-mouse probability prediction volumes; (2) assign each voxel to one class, based upon the maximum predicted probability; (3) apply morphological cleaning; and (4) resize the whole-mouse label map to the original whole-mouse input size. Step 1 is different for *2D-slices*, *3D-whole-mouse* and *3D-patches,* whereas steps 2–4 are the same for each model. In step 1 for *2D-slices*, since the predictions have the same number of slices as the original whole-mouse input, slice predictions are resampled to the original x–y dimension, then stacked to generate the volume prediction for each mouse. In step 1 for *3D-whole-mouse*, resampling in the x–y–z dimensions is performed, to match the original input size for each mouse. In step 1 for *3D-patches*, more processing is required to reconstruct predictions. Voxels in the center of a patch have predictions from only one patch; voxels at the periphery of patches have predictions from multiple overlapping patches. For a voxel in an overlapping region, the maximum predicted probability is assigned for each class. Since zero-padding is performed during the *3D-patches* generation process, the reconstructed *3D-patch*es predictions are cropped to the original whole-mouse input size. Step 2 aggregates the predicted probabilities from all classes and generates one class label for one voxel, using the maximum probability. In step 3, 2D hole-filling, and 3D closing (using a ball-shaped structuring element with radius of 1 and size of 3 × 3 × 3) are performed for each organ label. Additionally, we run 3D connected components and keep only the largest component.

## Experimental methods

### Cryo-imaging experiments

We used 71 mice in this project, from cryo-image mouse volumes acquired using the CryoViz imaging system (Bioinvision, Cleveland, OH, USA). The frozen mice were sectioned at 40-μm slice thickness and imaged at 10.472 × 10.472 μm in-plane resolution. Color images were obtained using a liquid crystal RGB filter and monochrome camera. The mice were of different ages and from several different studies; therefore, they had significant differences, especially in the lung, liver, spleen, and kidney. As a demonstration project, we evaluated liver and spleen size changes in mice with acute liver injury induced by carbon tetrachloride (CCl_4_) and in a MDR2-KO mouse model for chronic liver disease. For the acute liver injury study, there were 19 mice with disease treatment and 20 healthy controls, all aged 8-weeks old. The treated mice received an intraperitoneal CCl_4_ injection, then received Qtracker 605 beads (Thermo Fisher Scientific) labeled mesenchymal stem cells (MSCs) 3 h later^30^. Four hours after intravenous injection of MSCs, the mice were sacrificed using cardiac puncture under isoflurane anaesthesia for cryo-imaging. For the MDR2-KO mouse study, there were two male MDR2^−/−^ mice aged 6–8 weeks old and two healthy control FVB mice of the same age. We analyzed the distribution of MSCs in one mouse from an acute liver injury study. The MSCs were automatically detected using previously-developed in-house software^5^. The distribution was compared against manual segmentation performed by an expert in cryo-imaging. All procedures and animal housing conditions were in strict accordance with European Union legislation on animal experimentation and were approved by the Institutional Committee for Animal Research (DEC protocol EMC No. 127-12-14). The study was carried out in compliance with the ARRIVE guidelines.

### Computational experiments

Model training, hyperparameter optimization, and performance assessment were conducted in the training, validation, and testing sets, respectively. We built our CNN network using Keras Tensorflow software and used the Adam optimizer (exponential decay rate of 0.9 for the 1st moment estimates, and 0.999 for the 2nd moment estimates). We used an NVIDIA GeForce RTX 2080 Ti graphics card with 12 GB memory as the GPU. The 71 mice were split 63/4/4 for training, validation, and testing, respectively. During training, due to the limitation of the GPU memory, mini-batch sizes were fixed to 24, 2, and 5 for *2D-slices*, *3D-whole-mouse*, and *3D-patches*, respectively. For *2D-slices*, only slices with at least one voxel containing organs of interest was used for training and validation; for *3D-patches*, only patches with organs > 10% of the whole patch volume were used for training and validation. The learning rate was reduced by a factor of 10 if the validation loss did not decrease for 10 epochs. The stopping criteria were: no decrease in validation loss for 15 epochs and 200 epochs reached, whichever came first. The final model was the one that had the lowest validation loss. The hyperparameter used to tune all three models was the initial learning rate (10^−3^ to 10^−4^ with a step size of 2 × 10^−4^). To evaluate segmentation performance, the Dice score overlap coefficients and Hausdorff distances (HDs) were calculated against the annotations from Analyst 1. Specifically, given the ground truth manual annotation (X) and label of prediction (Y) for each organ, the Dice score overlap coefficients were computed by Eq. (1). HDs were computed by the following equations:

4 $${HD}_{i}=\mathrm{max}\left(h\left({BX}_{i},{BY}_{i}\right), h\left({BY}_{i},{BX}_{i}\right)\right), i=1:10,$$

$$h\left({BX}_{i},{BY}_{i}\right)={max}_{x\in {BX}_{i}}\left({min}_{y\in {BY}_{i}}\left(d\left(x,y\right)\right)\right),$$

$$h\left({BY}_{i},{BX}_{i}\right)={max}_{y\in {BY}_{i}}\left({min}_{x\in {BX}_{i}}\left(d\left(y, x\right)\right)\right),$$

where *BX*_*i*_ and *BY*_i_ are the boundary point sets in the manual annotation and the prediction label for organ i, respectively; d(x, y) is the Euclidean distance between x and y. If there was no prediction of an organ, the Dice score was zero and HD was set to 30 mm. A second analyst (Analyst 2) performed annotation of the testing set. We further compared the Dice scores and HDs of the three CNN models with the annotations from the Analyst 2. Analysts 1 and 2 performed editing of the segmentation results generated by the best performing CNN model on the same test set. The amount of editing time was recorded and Dice scores between the edited segmentations and between the annotations from two analysts were calculated.

To assess the generalizability of the deep learning model to a variety of unseen data, we performed cross validation. The training/validation/testing split was 63/4/4 for all three types of input. We randomly selected eight folds for calculation. Given the optimized hyperparameters, the validation dataset was used solely for stopping the training process, to avoid overfitting. We compared the results among *2D-slices*, *3D-patches,* and *3D-whole-mouse* models using the Friedman test. A p-value < 0.05 indicated a significant result. We used the Friedman test instead of ANOVA because it uses rank-transformed data and because the Dice scores and HDs for all organs do not follow a normal distribution.

To generate learning curves for the three types of input for each organ, we decreased the number of input mice from 71 to 15, with a step size of eight. The mice in the validation/test sets were fixed to be the same 4/4 mice across all experiments with different number of training samples among the three types of inputs. The number of training mice decreased from 63 to seven, with a step size of eight; mice were randomly selected in each training set. To predict the Dice scores of the three deep learning models with a large training set, we evaluated two regression models for learning curves using Eqs. (5)^29^ and (6)^28^. We used the Akaike information criterion corrected for a finite number of samples (AIC_c_) in Eq. (7)^31^ was applied to select the best performing model for each organ.

5 $$Dice=a\cdot \mathrm{exp}\left(bx\right)+c\cdot \mathrm{ln}\left(x+d\right)+e,$$

6 $$Dice=\left(1-a\right)-b\cdot {x}^{c},$$

7 $${AIC}_{c}=2p+2C+\frac{2p(p+1)}{n-p-1},$$

where *p* is the number of parameters, n = 8 is the number of data points for fitting the model, and *C* is the sum of squared errors. The best model for each organ was the one with the lowest AIC_c_. In predicting Dice scores with a large training set (> 63 mice), we expected that the Dice scores from 63 training mice is more indicative than Dice scores from 7 training mice. Therefore, instead of using linear regression^29^, we adopted nonlinear weighted least squares optimization, together with the nl2sol routine from Port Library in R^28^ for fitting both equations. The weight for each data point was calculated as *j/m* where *j* is the current number of training samples and *m* is the maximum number of training samples. The fitted models allowed us to predict the number of samples needed to achieve a Dice score of 0.9 for each organ.

Using an NVIDIA A40 graphics card with 48 GB RAM, we evaluated the effect of some hyperparameters that could be varied, prompted by reports claiming a dependence on input size^22^ and mini-batch size^32^. We investigated the effects of increasing mini-batch size for *2D-slices*, increasing input resolution for *3D-whole-mouse*, and compared larger mini-batch size vs. larger patch size for *3D-patches*. The median shape of all mice was 928 × 327 × 146. For *2D-slices*, the resampled input size (768 × 256) was close to the median shape; we only tested for larger mini-batch size. For *3D-whole-mouse*, each sample contained all organs of interest; a batch size of two was deemed reasonable^22^; therefore, we only tested for higher resolution, which is equivalent to larger input size. For *3D-patches*, both larger mini-batch size and larger patch input size enabled more organs of interest for training in one iteration; therefore, we tested for both conditions. The new mini-batch size for *2D-slices* was 96. The new input size for *3D-whole-mouse* was 576 × 192 × 128. The new mini-batch size with the original patch size of 288 × 96 × 48 was 16. The new patch input size was 320 × 192 × 96, with a mini-batch size for four, which is close enough to a mini-batch size of five. The learning rates were kept the same as in training with the NVIDIA GeForce RTX 2080 Ti graphics card.

## Results

### Segmentation performance

**Figure 2:**
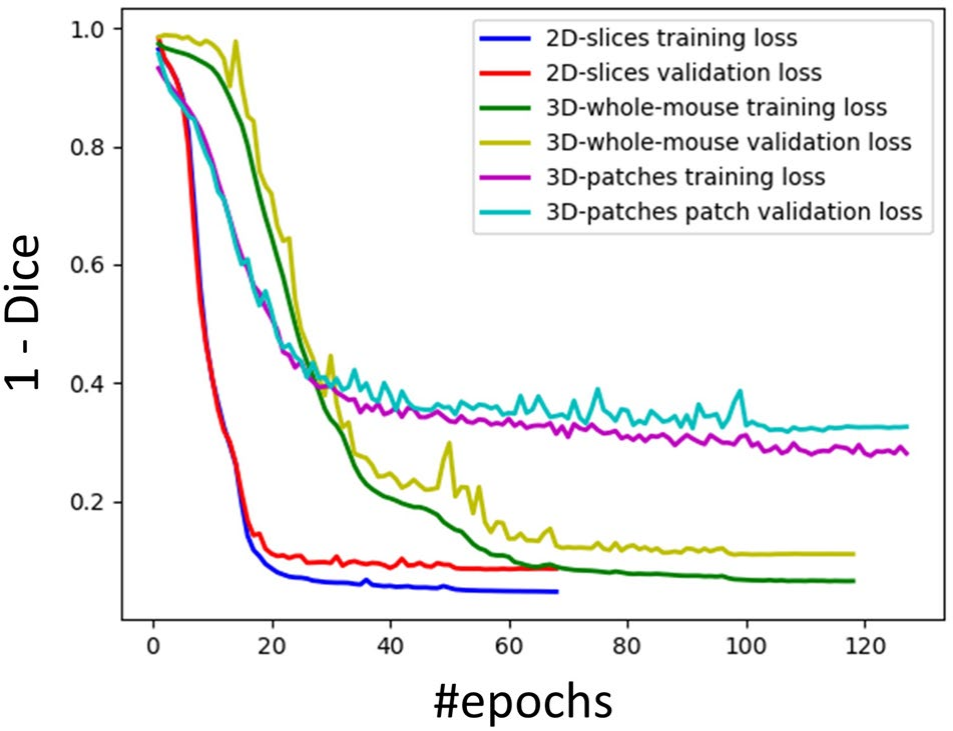
Training curves for *2D-slices*, *3D-whole-mouse*, and *3D-patches.* The graph shows training and validation loss changes with respect to the number of epochs.

We optimized the initial learning rate and generated optimized training curves using 63 training and four validation mice. The optimized initial learning rates were 1 × 10^−4^, 4 × 10^−4^, and 1 × 10^−4^ for *2D-slices*, *3D-whole-mouse*, and *3D-patches*, respectively. Training curves of the three CNNs with *2D-slices*, *3D-whole-mouse*, and *3D-patches* are shown in Fig. 2. The total numbers of epochs were 69, 119, and 124, respectively, for the three inputs. For *2D-slices*, the Dice losses decreased the most quickly and smoothly among the three CNNs. The validation loss closely followed the training loss before Epoch 16. For *3D-whole-mouse*, training loss was smooth, whereas validation loss fluctuated more than in the other two CNNs. The losses of *3D-whole-mouse* decreased more slowly than *3D-patches* during the first 10 epochs, but was faster after 10 epochs. For *3D-patches*, both training and validation losses fluctuated. The training and validation losses in *3D-patches* were much higher than the losses in *2D-slices* and *3D-whole-mouse*. This is because the batch normalization layer utilizes the running mean and standard deviation from the current mini-batch; our mini-batch size was too small to correctly represent the whole population. However, more importantly, the test performance of *3D-patches* was comparable to the other two models, as shown below.

**Figure 3:**
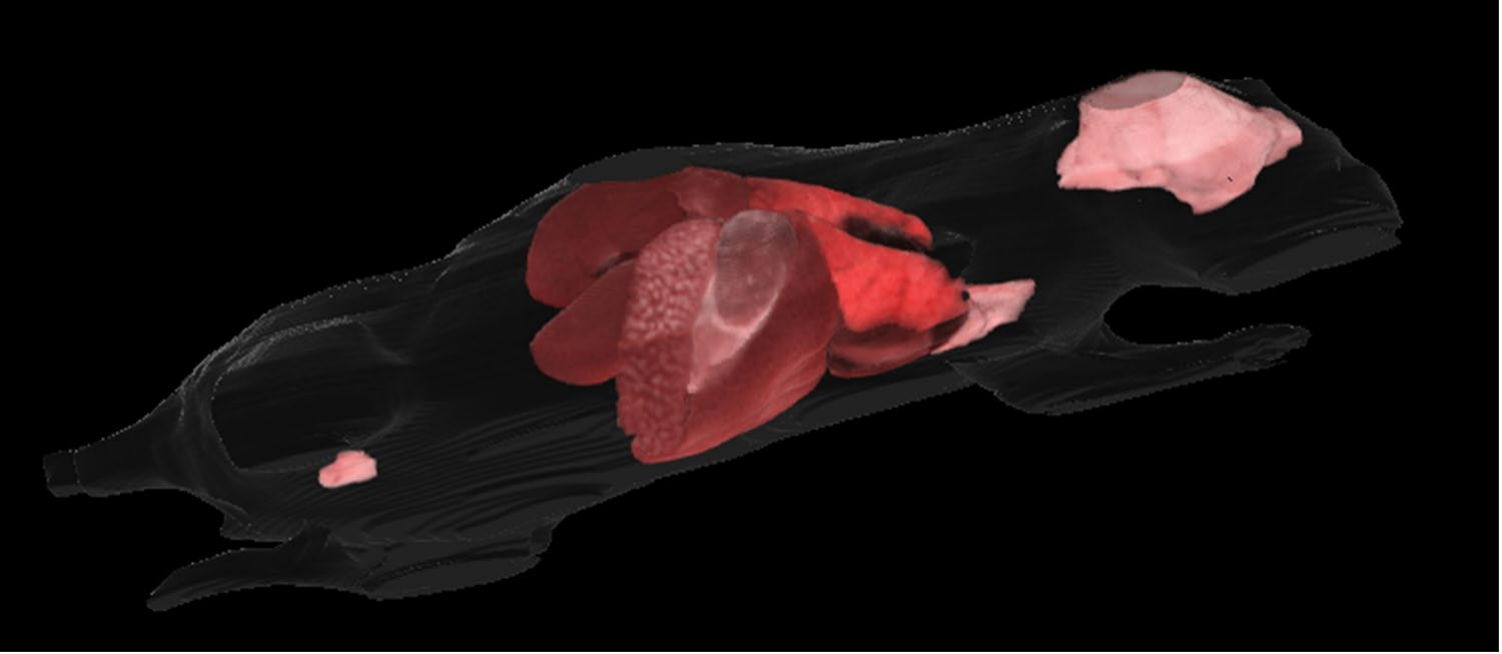
3D true color volume rendering of segmented mouse organs from *2D-slices* results. The organs are volume rendered and the mouse body label is surface rendered. The organs have various textures and colors.

**Figure 4:**
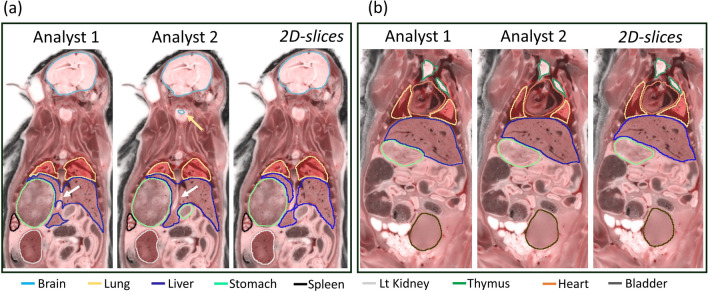
2D visualization of segmentation results from the *2D-slices* model and annotations from Analysts 1 and 2. Both analysts mistakenly included esophageal tissue (white arrows). Analyst 2 mistakenly labeled some spinal cord as brain (yellow arrow in (**a**)). Good correspondence between the two analysts and the *2D-slices* model is shown in (**b**).

**Figure 5:**
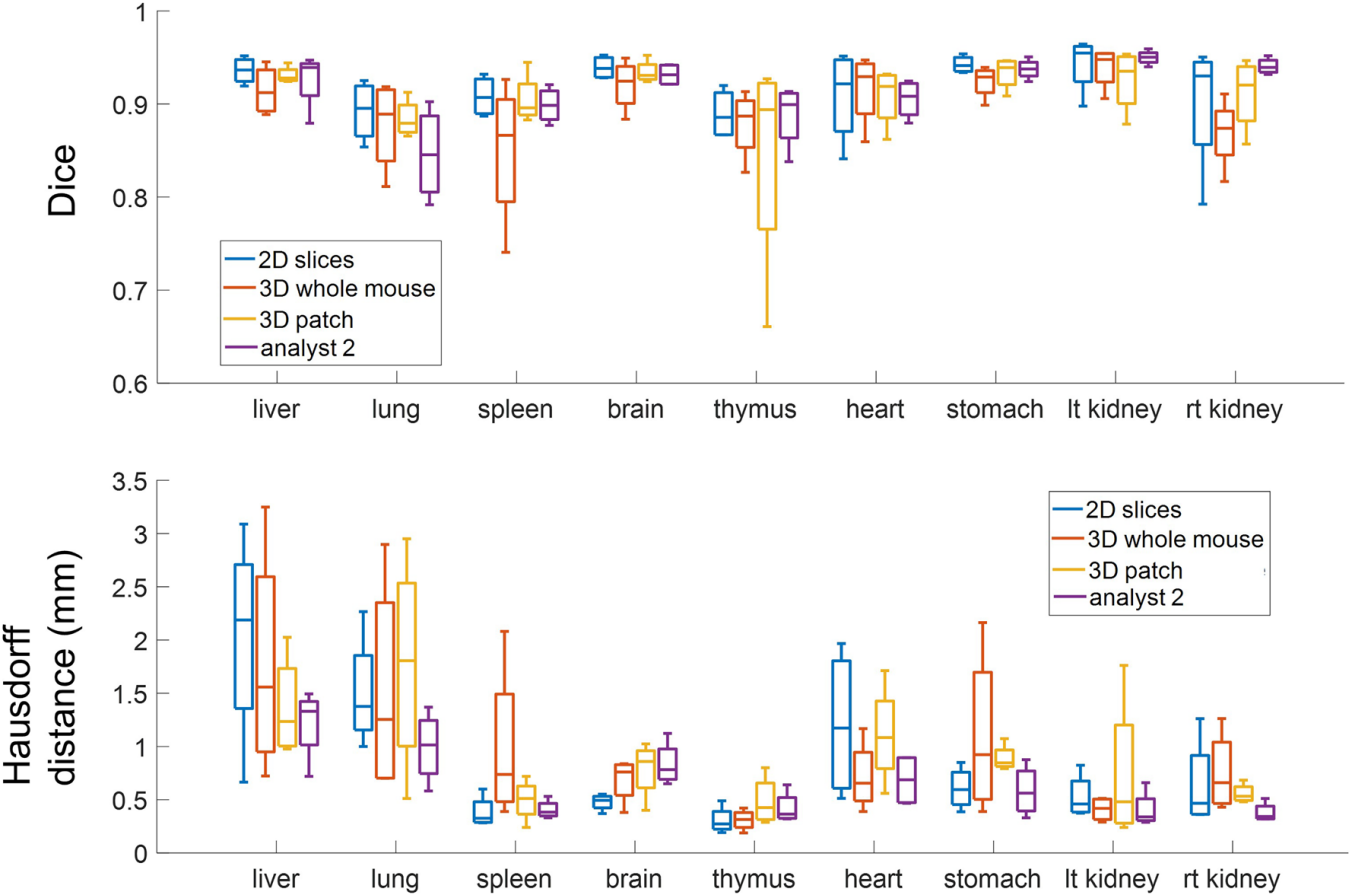
Performance of the three networks, and Analyst 2, against the manual segmentation of Analyst 1. (Top) Dice scores for the three proposed methods against the annotations of Analyst 1 (the ground truth), for considered organs, along with Dice scores for Analyst 2, as compared to Analyst 1. (Bottom) Similar plots for Hausdorff distance. The median Dice scores and Hausdorff distances for the best method, *2D-slices*, were similar to those for Analyst 2, compared against Analyst 1. Refer to the text for a statistical analysis.

We continued with this experiment by testing the segmentation performance of the three CNNs on four test mice against the annotations of Analyst 1 (the more experienced analyst), as the ground truth. We also evaluated Analyst 2 against Analyst 1, to assess potential variability among experts. With the segmentation results from *2D-slices*, true color 3D volume rendering of the segmented organs is shown in Fig. 3; surface rendering of the segmentation labels is shown in Supplemental Movie 1. Two-dimensional visual comparisons of the results from the *2D-slices* model and the annotations from Analysts 1 and 2 are shown in Fig. 4. We observed that both analysts mistakenly included some esophageal tissue (indicated by white arrows) and that Analyst 2 mistakenly labeled some spinal cord as brain, indicated by a yellow arrow in panel (a). In this instance, deep learning performed a better segmentation than the two analysts. Panel (b) shows good correspondence between Analysts 1, Analyst 2, and *2D-slices* segmentation. Quantitative Dice scores and HD results are shown in Fig. 5 and Tables S1 and S2. We omitted the bladder in Fig. 5 because it had poor Dice scores and HD values that changed the dynamic range of figures. Among the three CNN models, *2D-slices* had the best median Dice score in all organs, except the thymus and heart. It had the best median HD for the spleen, brain, thymus, stomach, and left and right kidneys. The median Dice scores of Analyst 2 were better than *2D-slices* for the liver, thymus and right kidney. Analyst 2 had the best median HD in the lung, stomach, and left and right kidneys. A comparison between *2D-slices* and Analyst 2, using the Wilcoxon signed-rank test, found no significant differences in Dice score or HD, across all organs. The Dice scores for the bladder were 0.6244 ± 0.3723, 0.4454 ± 0.4556, 0.2277 ± 0.2052, and 0.8534 ± 0.0633 for *2D-slices*, *3D-whole-mouse*, *3D-patches*, and Analyst 2, respectively (considering Analyst 1 as the ground truth). To improve bladder segmentation, we used a 2-step process. We used predictions from *2D-slices* to generate a bounding box for a localized field of view, resampled the input bounding box to 64 × 64 × 16, and fed it into a 3D U-Net with depth of 3 for training in the second stage. The Dice score on the test set was 0.8116 ± 0.0265, which is greatly improved as compared to the original multi-organ segmentation results from the three models.

**Figure 6:**
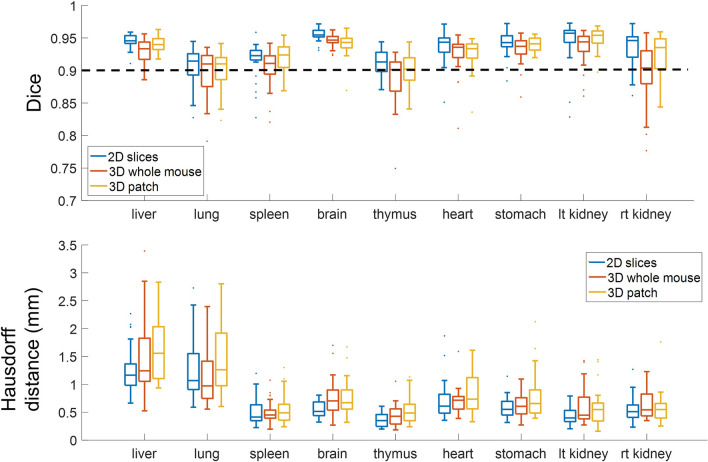
Comparison of the three models for an eightfold cross validation across organs. Dice scores (top) and Hausdorff distances (bottom) are shown. *2D-slices* had best median Dice score and Hausdorff distance across a majority of organs, and was significantly different from the other two models.

Using the learning rates assigned in experiments above, we evaluated the generalizability of the three models to a variety of unseen data, using eightfold cross validation. Quantitative Dice scores and HDs from eightfold cross validation are shown in Fig. 6 and Tables S3 and S4. Bladders were excluded, as described above. Across all organs, all three models had median Dice scores greater than or equal to 0.9. *2D-slices* had the best median Dice score and HD, except in the lung, where *3D-whole-mouse* gave a better median HD. From the Friedman test, the Dice score of *2D-slices* was significantly different from those of *3D-whole-mouse* and *3D-patches* (p < 0.02). *3D-whole-mouse* was not significantly different from *3D-patches* (p > 0.1). For HD, only *2D-slices* and *3D-patches* were significantly different. The Dice scores of the bladder were 0.6419 ± 0.2649, 0.6470 ± 0.2938, and 0.3223 ± 0.3593 for *2D-slices*, *3D-whole-mouse*, and *3D-patches*, respectively, without the two-step process.

### Labeled data efficiency and sample size planning

**Figure 7:**
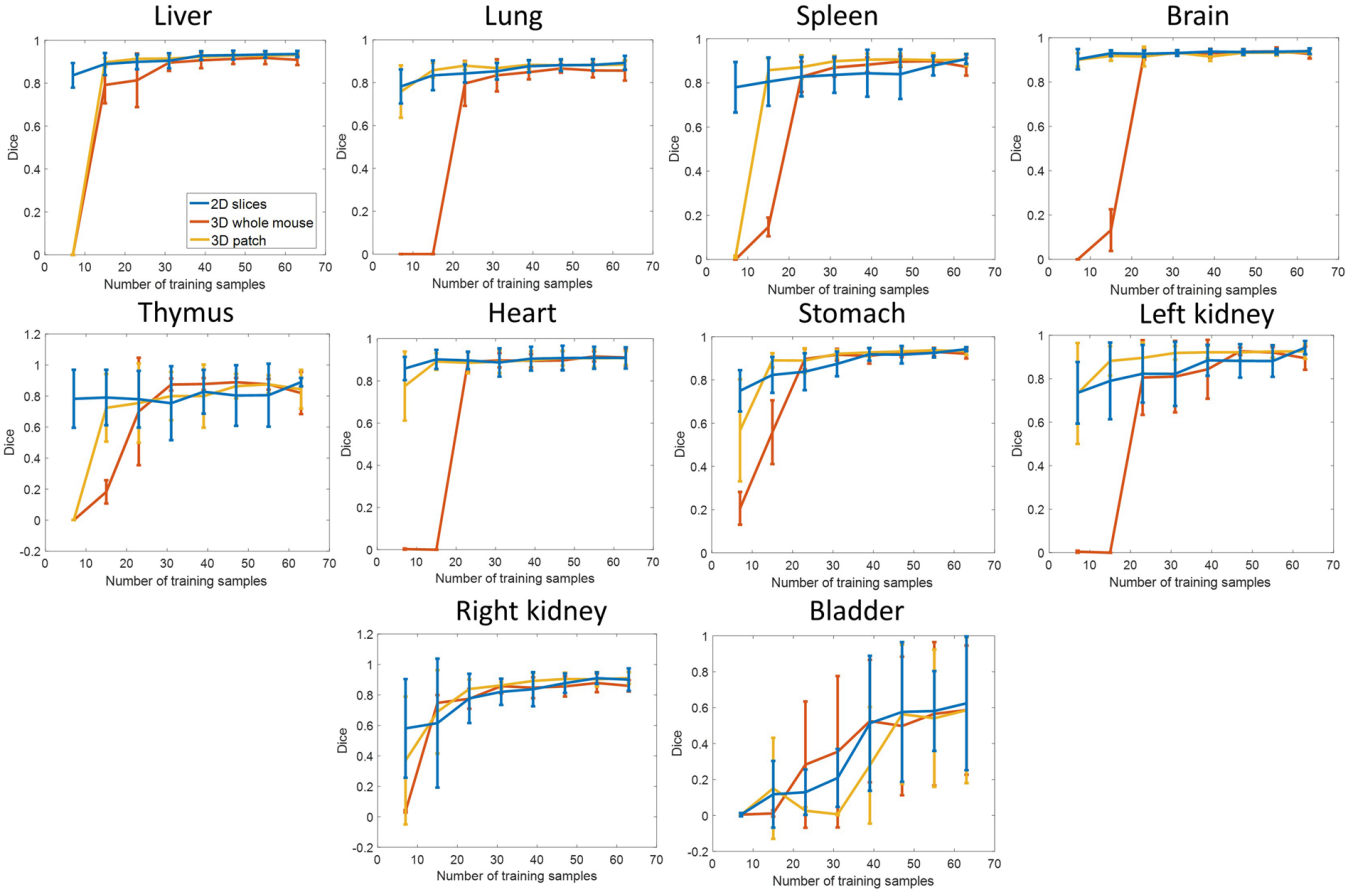
Learning curves showing Dice scores against numbers of training data for the three models, across all organs. *2D-slices* had the best performance, given a small training dataset with seven mice. Regression analyses were performed to predict the Dice score given a larger training dataset (see text for details).

Learning curves of Dice scores against the number of training samples for each organ are shown in Fig. 7. Learning curves help us to understand which model works best with a small amount of training data and identify the performance gain with more training data. The figure shows data for the bladder without the two-step process, but we focused on the results of the other nine organs for comparisons of the CNN models. Since we randomly picked training, validation, and test data, there are fluctuations in the learning curves. With seven mice for training, *2D-slices* had the best Dice score among the three models across all organs. For *3D-whole-mouse*, it required at least 23 training samples for all organs to have non-zero Dice scores. For *3D-patches*, the best average Dice scores were for the spleen and left and right kidney when the number of training samples was in the range of 23–39. With 63 training samples, the Dice scores of all organs reached 0.9, except the thymus in *2D-slices.* The Dice scores of the liver, brain, heart, stomach, and left kidney reached 0.9 in *3D-whole-mouse.* All organs except the lung and thymus reached a Dice score of 0.9 in *3D-patches*. The liver, brain, and heart were easy to segment as the Dice scores with 15 training samples were close to the Dice scores with 63 training samples. By fitting the learning curves to Eqs. (5) and (6), we predicted the minimum number of training samples required for each organ (except the bladder) for a Dice score of 0.9. The numbers of mice needed for *2D-slices*, *3D-whole-mouse*, and *3D-patches* were 88, 87, and > 1000 with thymus, right kidney, and lung being the most challenging organs, respectively. The reason why the lung in *3D-patches* required > 1000 samples is that the learning curve plateaus at a sample size of 39, with a Dice score of ~ 0.88.

### Analysts editing of the CNN segmentation result

**Figure 8:**
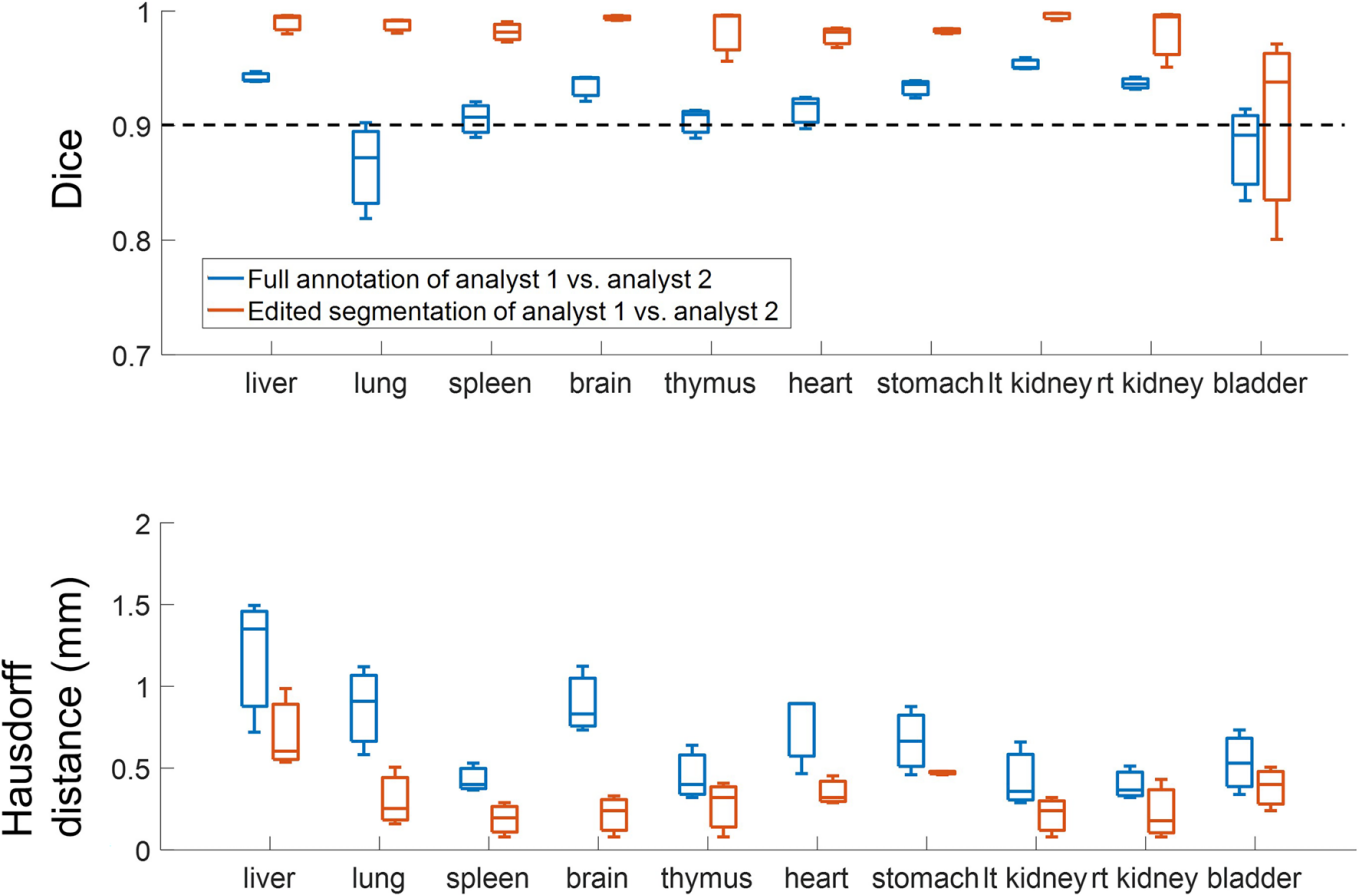
Comparison of Analyst 2 versus the more experienced Analyst 1. Comparison of Analyst 2 versus Analyst 1 with manual segmentation (blue) and with editing of deep learning segmentation (red). Ten organs were evaluated in three mice. Dice scores improved for each organ with editing of the deep learning result, as compared with fully manual segmentation (top). The HDs were similarly improved, with lower values indicating improved segmentation (bottom). In addition to improved reproducibility, the editing approach greatly reduced editing time, compared with a fully manual analysis (see text).

**Figure 9:**
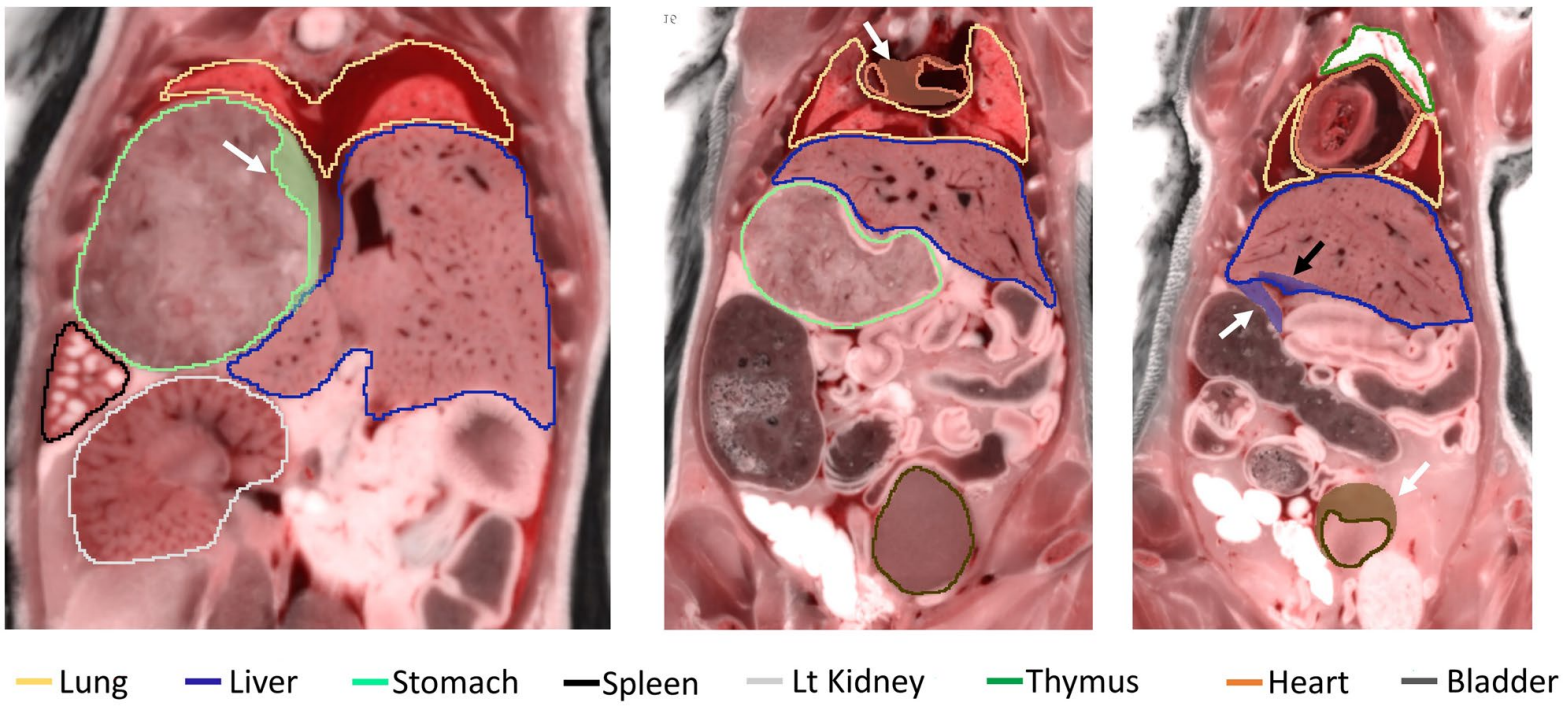
Examples showing analysts’ edits of various organs. Predicted segmentations are outlined with solid lines. Edits are shown as transparent shaded regions. Analysts added regions to the stomach, heart, liver, and bladder (white arrows) and removed regions in the liver (black arrow).

We evaluated the one stage *2D-slices* method for routine use, using manual review and editing. We analyzed both editing time and inter-observer differences. For three mice with deep learning segmentations, the editing times were 19 ± 10 min and 30 ± 30 min for Analysts 1 and 2, respectively. For comparison, manual editing typically takes > 2 h. To examine the effect on inter-observer differences, we compared Analyst 2 to the more experienced Analyst 1, with and without prior automated segmentation (Fig. 8). Both the median Dice score and HD improved for each of 10 organs in the case of editing of automated segmentation, as compared to the fully manual approach. The difference was highly significant (Wilcoxon signed-rank test: p < 0.001). Assuming Analyst 1 to be the ground truth, the accuracy of Analyst 2 improved when given an automated segmentation to edit. Some example edits are shown in Fig. 9. Most edits occurred in slices near the ventral and dorsal surfaces.

### Application of organ segmentation

**Figure 10:**
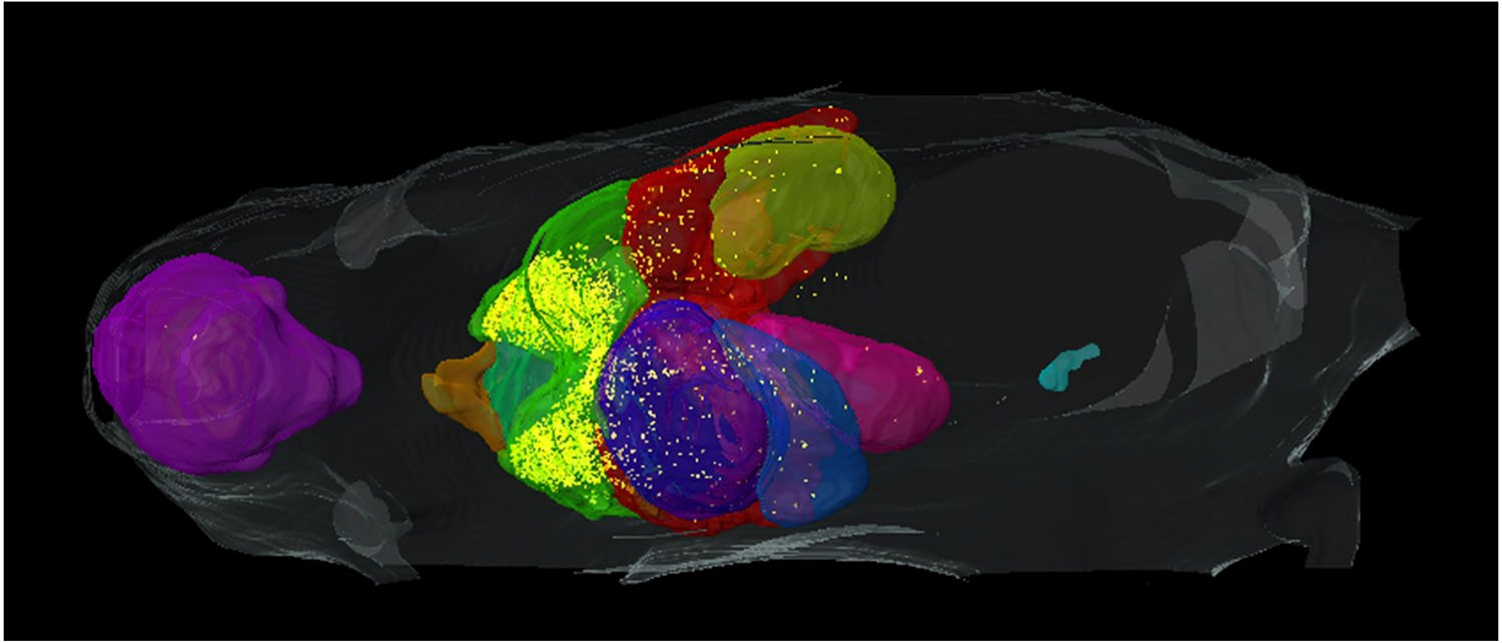
3D visualization of MSC distribution in a mouse with acute liver injury induced by CCl4. Detected MSCs are surface rendered in yellow. There were 22,797 MSCs, with 19,656 in the lung, 2082 in the liver, 70 in the spleen, seven in the heart, 12 in the left kidney, two in the right kidney, none in the brain, stomach, thymus, or bladder, and 968 in the rest of the mouse body. The quantifications based upon manual segmentations were: 18,935 in the lung, 2081 in the liver, and 67 in the spleen, giving a smaller than 5% disagreement. Our fully automated analysis provides a means for quick and thorough analysis of distribution to a much larger number of organs than is practical with manual segmentation.

We demonstrated the application of our automated segmentation method in experiments with liver conditions. We analyzed an experiment to determine the biodistribution of therapeutic MSCs in an acute liver injury model induced by CCl4 (Fig. 10). It has been shown previously that intravenously injected MSCs are mostly found in the lung, liver, intestine, skin, bone marrow, and spleen^33^. The majority of the MSCs in our experiment (86.2%) were found in the lung. Quantification using our automated segmentation method (*2D-slices)* gave results within 5% of manually obtained results for the lung, liver, and spleen. Our fully automated analysis provides a means for quick and thorough analysis of distribution to a much larger number of organs than is practical with manual segmentations (see figure legend).

We demonstrated organ volume measurements in our experiments. First, we evaluated liver and spleen volume differences in different liver conditions. For an acute liver injury model, there were no significant changes. Liver volumes in the affected and control groups were 1.50 ± 0.26 cm^3^ and 1.53 ± 0.26 cm^3^, respectively; spleen volumes were 85.55 ± 22.08 mm^3^ and 80.60 ± 21.56 mm^3^, respectively. Although treatment with CCl4 induces oxidative damage, inflammation, fatty degeneration, and fibrosis in the liver^34^, the mice were exposed for only a short period of time; therefore, volumes did not significantly decrease. Second, we evaluated the MDR2-KO mouse, which spontaneously exhibits primary sclerosing cholangitis (PSC), a chronic liver disease; with targeted disruption of the multi-drug resistance gene 2 (*Abcb4*). An absence of biliary phospholipids leads to the leakage of bile acids and cytotoxic injury to the biliary tree. An increase in the volumes of the spleen and left lobe of the liver in human primary sclerosing cholangitis is indicative of adverse events^35^. For the MDR2-KO mice and controls, liver volumes were 1.63 ± 0.31 cm^3^ and 1.47 ± 0.30 cm^3^, respectively, which is an increase of around 11%. Spleen volumes were 110.68 ± 30.82 mm^3^ and 96.19 ± 41.23 mm^3^, respectively. For chronic liver disease, there was a strong trend toward larger volumes of both the liver and spleen; however, the changes were not significant.

### Training with large-memory GPU

Using the NVIDIA A40 GPU, we explored the impact on deep learning of its 48 GB RAM (Supplementary Materials). While some have argued about the role of small mini-batch size in regularization^32^, larger mini-batch sizes allow faster computation and more accurate gradient estimation. We varied mini-batch size from 24 to 96 slices, for *2D-slices,* and compared the accuracy of the results (Supplementary Fig. S1). There was no significant difference in Dice scores between 96 and 24 slices (Wilcoxon signed-rank test: p > 0.1). It has been shown in nnU-Net that input patch size is important in training and they used a patch size of at least 25% of the median image shape. We varied the patch size for *3D-patches* from 288 × 96 × 48 (3% of median shape) to 320 × 192 × 96 (13% of median shape). A comparison between a larger patch size with a larger mini-batch size of 16 and the original small patch size with a mini-batch size of five found no significant differences in Dice scores (Friedman test: p > 0.05) (Fig. S2). Therefore, a patch size of 288 × 96 × 48 (being ~ 1/3 of each dimension of the whole-mouse) and a mini-batch size of five are good enough to train the network. Finally, we increased the input size for *3D-whole-mouse* which is equivalent to improving input resolution, and found improvement in the median Dice scores for all organs except the heart, and found reduced median HDs of the spleen, brain, heart, stomach, and left and right kidneys (Fig. S3). There was a significant difference in Dice scores between low- and high-resolution input (Wilcoxon signed-rank test: p < 0.001). Thus, if more memory is available, the best way to improve segmentation performance is to increase input resolution*.*

## Discussion

Deep learning-based multi-organ segmentation is an important addition to cryo-imaging analysis software for studying many biotechnology applications (e.g., stem cell therapy and metastatic cancer therapies). The *2D-slices* model offers good segmentation performance with eightfold cross validation, as indicated by Dice scores > 0.9 (Fig. 6). It takes a human analyst ~ 2 h to segment the 10 target organs in a whole-mouse. It takes only about 25 min to review, and possibly edit, the deep learning segmentation results. In some instances, this may result in improved segmentation (see Fig. 4, for example). Aside from being time-consuming, manual annotation may introduce interpolation errors when using the semi-automatic annotation software, such as 3DSlicer, as shown in Fig. S4.

Training curves provide us with important insights for explaining the segmentation performance of the three CNNs. The *2D-slices* model converged fastest among the three models, likely due to 2D U-Net having few parameters and more training samples from the 2D slices input. The validation curve follows the training curve closely at the early phase of training for both *2D-slices* and *3D-patches*. This demonstrates that the trained models generalized well to the validation dataset. For *3D-whole-mouse*, the validation loss fluctuated and was higher than the training loss at the early phase of training. This difference could be due to the fact that *2D-slices* and *3D-patches* offer more training samples than *3D-whole-mouse*, which improves the generalizability of these CNNs at an early phase. When all three CNNs converged, the training loss of *3D-whole-mouse* was close to that of *2D-slices*, and the difference between training and validation loss was similar to the other two CNNs. This indicated that, although the number of *3D-whole-mouse* inputs was significantly less than in *2D-slices* or *3D-patches*, it was enough to make 3D U-Net converge to being as good as both 2D U-Net and 3D U-Net with *3D-patches* input. The training loss of *3D-patches* fluctuated after Epoch 30, whereas the training loss of *2D-slices* and *3D-whole mouse* were smooth; this indicates the instability of the *3D-patches* model.

The learning curves (Fig. 7) demonstrate that the *2D-slices* model is the best for a small number of training samples. However, based upon the analysts’ editing results, errors with *2D-slices* tended to occur at the top and bottom of organs, which suggests that additional 3D information is desirable. With more data, *3D-whole-mouse* could more closely approach the performance of *2D-slices*. Given a desired Dice score of at least 0.9 for all organs except the bladder, the training data size predicted for the *3D-whole-mouse* model was 87 samples. However, a downsampled whole-mouse input is not suitable for segmenting small tissues. It was found that small, thin organs, such as arteries, would benefit from the two-stage cascaded approach^13^. We have demonstrated improved segmentation performance on bladder using the two-stage method. In the second stage, 3D U-Net was used, as it performed better than 2D U-Net. We conclude that the best way to segment small tissues in cryo-images is a two-stage approach, including a first stage to identify the location, followed by refinement in high-resolution local volumes with 3D U-Net.

There are limitations and possible improvements to our methodology. The bladder is a challenging organ to segment because its shape varies greatly from mouse to mouse and contrast between the bladder and surrounding tissues is low. More data that provides a full representation of shapes and, potentially, a two-stage approach could improve segmentation performance for the bladder. We applied fast post-processing approaches (“Post-processing” section) that successfully cleaned hard segmentation results. Although *3D conditional random field* is an alternative, it is computationally very demanding; we selected the faster option.

In summary, the *2D-slices* deep learning model worked best for the segmentation of organs in our cryo-imaging color dataset; the segmentation results are promising. Deep learning improves the efficiency, accuracy, and robustness of automatic multi-organ segmentation, compared with human analysts. Segmented organs enable many downstream evaluations, such as quantifying the organ volumes in different disease models and the distribution of fluorescent MSCs or metastases in various organs.

## Supplementary Information

Supplementary Information.

Supplementary Legends.

Supplementary Movie 1.

## Data Availability

The datasets generated and/or analyzed during the current study are available for download through password-protected links below. Passwords may be obtained from DW or MG upon request for non-commercial use. Image data in .NII format is available for download at: https://www.dropbox.com/sh/i3v517zc68dbooq/AAA3n0a9po0Wl5bJo1X1vIq1a?dl=0. Python source code for 2D Unet is available for download at: https://www.dropbox.com/sh/9xloj0fdqlzgj63/AABkYrfpsRYTBnFdfGiIOcjPa?dl=0.
