# Supplementary material for: Deep learning multi-organ segmentation for whole mouse cryo-images including a comparison of 2D and 3D deep networks
Source: Sci Rep. 2022 Sep 7;12:15161. doi: 10.1038/s41598-022-19037-3 (PMC9452525; doi:10.1038/s41598-022-19037-3)
Supplement: Supplementary file 1 — Supplementary Information. [file 41598_2022_19037_MOESM1_ESM.docx]

**Deep learning multi-organ segmentation for whole mouse cryo-images including a comparison of 2D and 3D deep networks**

Yiqiao Liu^a^, Madhusudhana Gargesha^b^, Bryan Scott^b^, Arthure Olivia Tchilibou Wane^a^, David L. Wilson^a,b,c,*^

^a^Case Western Reserve University, Department of Biomedical Engineering, 10900 Euclid Avenue, Cleveland, Ohio, 44106, USA

^b^BioInVision Inc, Suite E 781 Beta Drive, Cleveland, Ohio, 44143, USA

^c^Case Western Reserve University, Department of Radiology, 10900 Euclid Avenue, Cleveland, OH, 44106, USA

* [dlw@case.edu](mailto:dlw@case.edu)

This supplemental material includes multiple figures and tables of interest. These are discussed in more detail in the main text; we simply provide an overview here. In Fig. S1, we show the effect of increasing mini-batch size on segmentation accuracy, by comparing mini-batch sizes of 24 slices and 96 slices in the *2D-slices* model. In Fig. S2, we show the effect of increasing mini-batch size and input patch size on segmentation accuracy. Three configurations were compared for the *3D-patches* model: input size 288 × 96 × 48 with mini-batch size of 5, input size 288 × 96 × 48 with mini-batch size of 16, and input size 320 × 192 × 96 with mini-batch size of 4. In Fig. S3, we show the effect of increasing input resolution on segmentation accuracy. For *3D-whole-mouse,* increasing input size is equivalent to improving input resolution. We compared input sizes 384 × 128 × 64 and 576 × 192 × 128. In Fig. S4, we show interpolation errors from human annotation of the liver, heart, and brain. We show the Dice scores and HD from *2D-slices*, *3D-whole-mouse*, *3D-patches*, and Analyst 2 on one test set in Table S1 and S2, respectively. We show the Dice scores and HD from *2D-slices*, *3D-whole-mouse*, and *3D-patches* in 8-fold cross validation in Table S3 and S4, respectively.


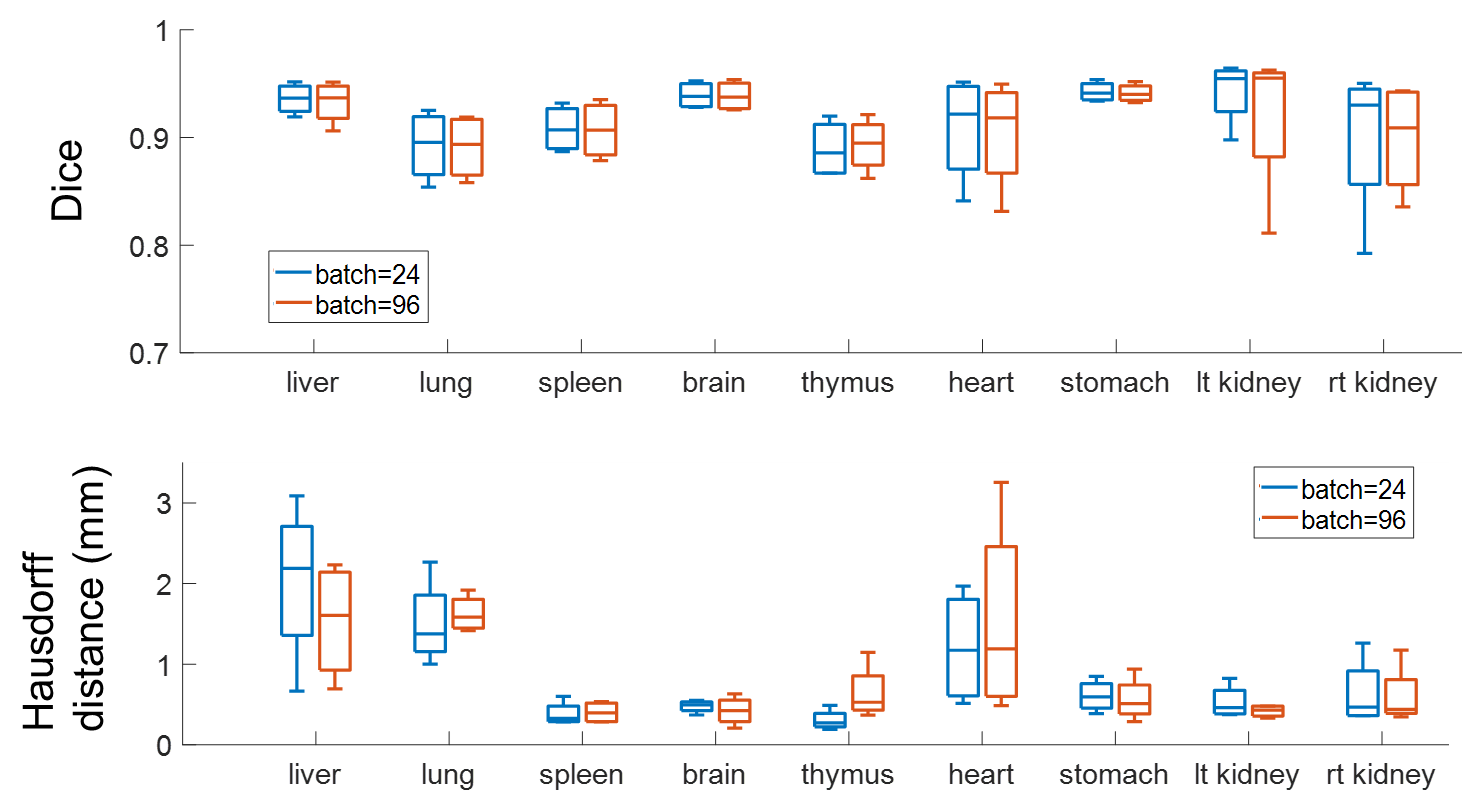


**Figure S1.** Effect of increasing mini-batch size on segmentation accuracy. We compared mini-batch sizes of 24 slices and 96 slices in the *2D-slices* model. A mini-batch size of 96 slices and 2D U-Net almost maximized the use of 48 GB of graphics processing unit memory. A mini-batch size of 24 slices and 2D U-Net almost maximized the use of 11 GB of graphics processing unit memory. There was no significant difference between 96 slices and 24 slices.


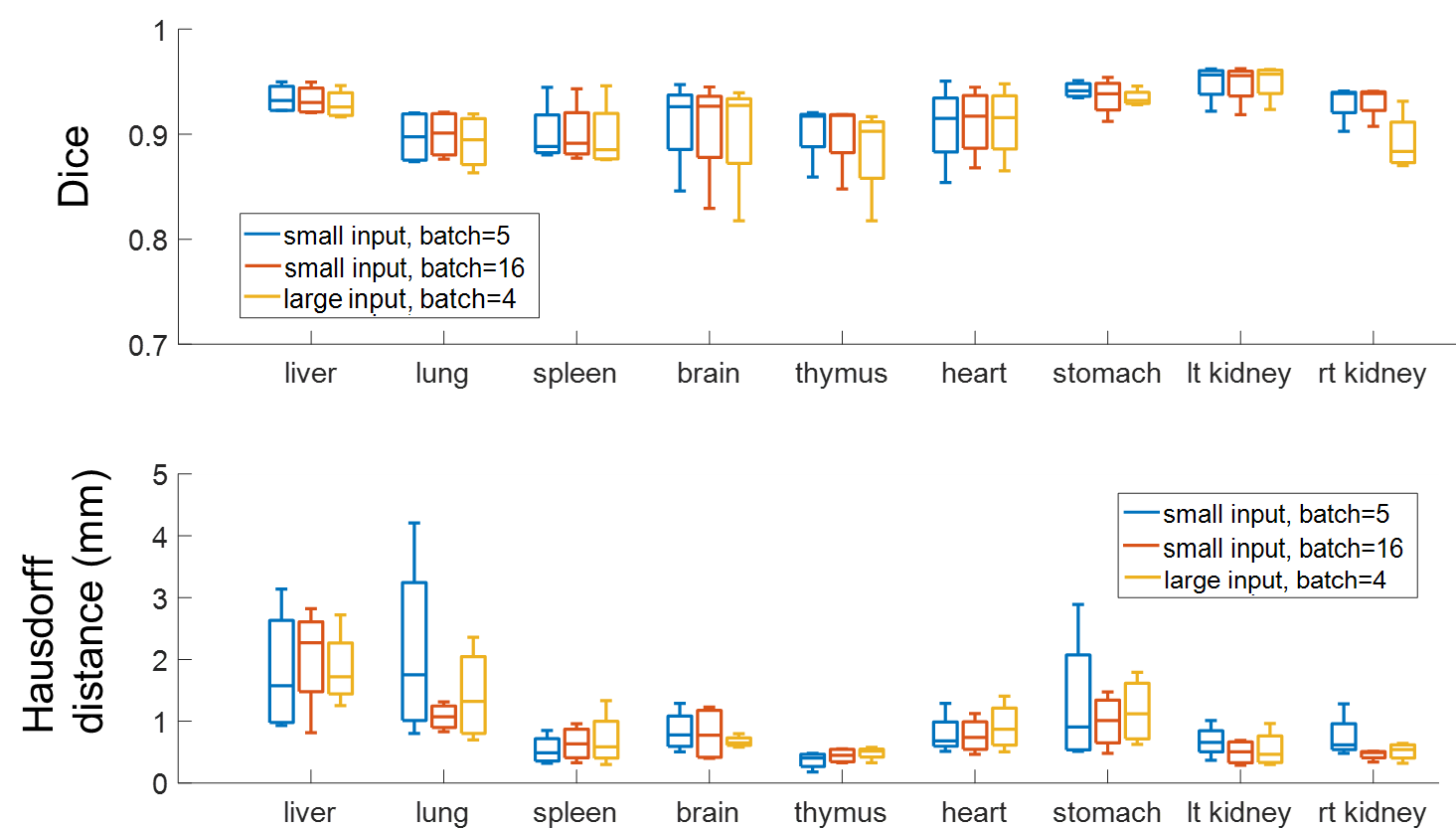


**Figure S2.** Effect of increasing mini-batch size and input patch size on segmentation accuracy. Three configurations were compared for *3D-patches*: input size 288×96×48 with mini-batch size of 5, input size 288×96×48 with mini-batch size of 16, and input size 320×192×96 with mini-batch size of 4. The first configuration and 3D U-Net nearly maximized the use of 11 Gb of graphics processing unit memory. The second and third configurations with 3D U-Net almost maximized the use of 48 Gb of graphics processing unit memory. There were no significant differences.


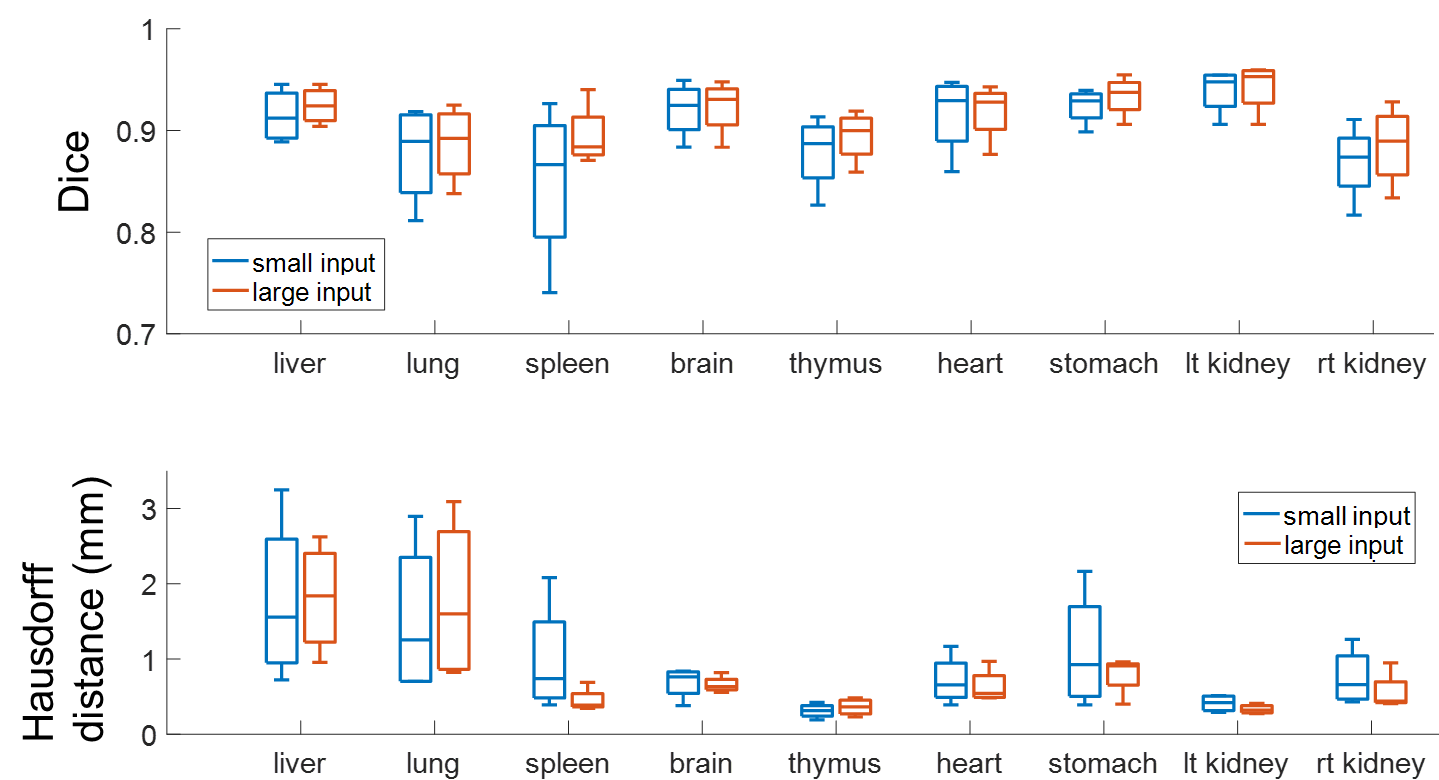


**Figure S3.** Effect of increasing input resolution on segmentation accuracy. For the *3D-whole-mouse* model*,* increasing input size is equivalent to improving input resolution. We compared input sizes 384×128×64 and 576×192×128. An input size of 576×192×128, mini-batch size of 2 volumes, and 3D U-Net almost maximized the use of 48 Gb of graphics processing unit memory. An input size of 384×128×64, mini-batch size of 2 volumes, and 3D U-Net almost maximized the use of 11 Gb of graphics processing unit memory. There was significant difference between the two input sizes. Median Dice scores improved for all organs, except the heart.


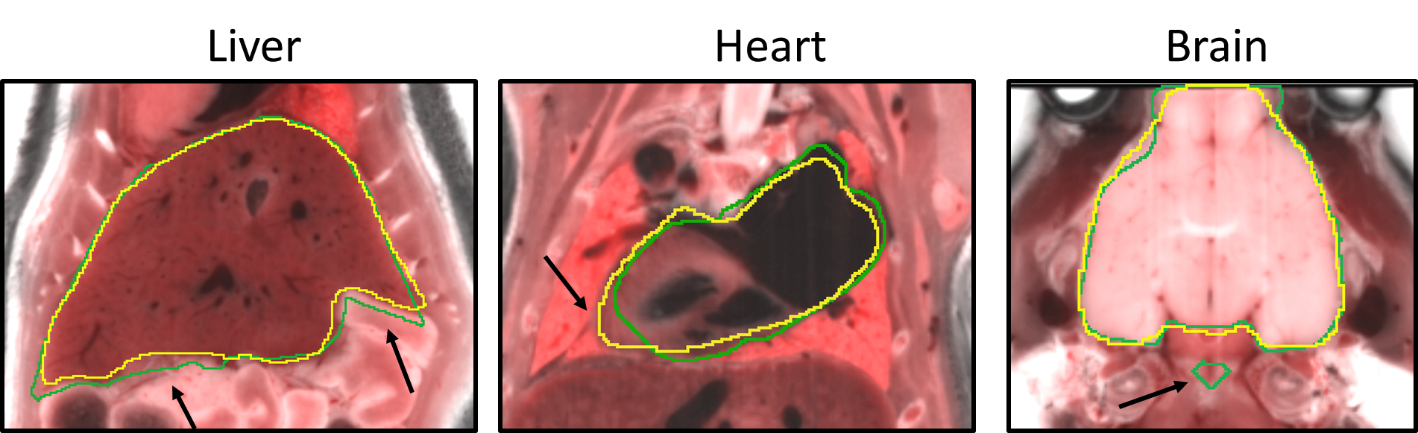


**Figure S4.** Annotations performed by analysts, with interpolation errors shown in the liver, heart, and brain. The green lines are human annotation. The yellow lines are the segmentation results from the *2D-slices* model. Black arrows point to where interpolation errors arose. In the liver, human annotations mistakenly included the gastrointestinal tract; in the heart, the apex of the heart was missing; in the brain, bone was mistakenly included.

Table S1. Dice scores of *2D-slices*, *3D-whole-mouse*, *3D-patches*, and Analyst 2, against the manual segmentation of Analyst 1.

|  | **2D-slices** | **3D-whole-mouse** | **3D-patches** | **Analyst 2** |
| --- | --- | --- | --- | --- |
| **Liver** | 0.9360±0.0145 | 0.9146±0.0267 | 0.9311±0.0091 | 0.9262±0.0315 |
| **Lung** | 0.8925±0.0329 | 0.8771±0.0496 | 0.8842±0.0205 | 0.8463±0.0502 |
| **Spleen** | 0.9083±0.0220 | 0.8499±0.0794 | 0.9048±0.0274 | 0.8987±0.0193 |
| **Brain** | 0.9393±0.0125 | 0.9205±0.0278 | 0.9345±0.0124 | 0.9316±0.0118 |
| **Thymus** | 0.8896±0.0269 | 0.8885±0.0372 | 0.8439±0.1247 | 0.8875±0.0347 |
| **Heart** | 0.9090±0.0506 | 0.9164±0.0397 | 0.9080±0.0325 | 0.9053±0.0209 |
| **Stomach** | 0.9426±0.0092 | 0.9240±0.0178 | 0.9334±0.0176 | 0.9375±0.0109 |
| **Lt kidney** | 0.9429±0.0307 | 0.9390±0.0229 | 0.9256±0.0343 | 0.9499±0.0079 |
| **Rt kidney** | 0.9007±0.0733 | 0.8688±0.0388 | 0.9111±0.0397 | 0.9405±0.0087 |
| **Bladder** | 0.6244±0.3723 | 0.4454±0.4556 | 0.2277±0.2052 | 0.8534±0.0633 |

Table S2. HD of *2D-slices*, *3D-whole-mouse*, *3D-patches*, and Analyst 2, against the manual segmentation of Analyst 1.

|  | **2D-slices** | **3D-whole-mouse** | **3D-patches** | **Analyst 2** |
| --- | --- | --- | --- | --- |
| **Liver** | 2.0325±1.0120 | 1.7719±1.1055 | 1.3682±0.4846 | 1.2187±0.3418 |
| **Lung** | 1.5049±0.5406 | 1.5265±1.0511 | 1.7691±1.0287 | 0.9951±0.3334 |
| **Spleen** | 0.3839±0.1480 | 0.9865±0.7599 | 0.4958±0.1979 | 0.4068±0.0874 |
| **Brain** | 0.4775±0.0781 | 0.6851±0.2120 | 0.7856±0.2702 | 0.8343±0.2061 |
| **Thymus** | 0.3064±0.1286 | 0.3098±0.0971 | 0.4850±0.2308 | 0.4225±0.1493 |
| **Heart** | 1.2062±0.7080 | 0.7167±0.3303 | 1.1098±0.4738 | 0.6838±0.2432 |
| **Stomach** | 0.6064±0.1987 | 1.1001±0.7934 | 0.8896±0.1258 | 0.5826±0.2395 |
| **Lt kidney** | 0.5299±0.2075 | 0.4102±0.1124 | 0.7405±0.7025 | 0.4065±0.1712 |
| **Rt kidney** | 0.6391±0.4262 | 0.7527±0.3797 | 0.5574±0.0905 | 0.3797±0.0910 |
| **Bladder** | 0.6874±0.5909 | 7.9550±14.6979 | 1.5700±1.5611 | 0.5841±0.1890 |

Table S3. Dice scores of *2D-slices*, *3D-whole-mouse*, and *3D-patches*, against the manual segmentation of Analyst 1.

|  | **2D-slices** | **3D-whole-mouse** | **3D-patches** |
| --- | --- | --- | --- |
| **Liver** | 0.9456±0.0106 | 0.9304±0.0182 | 0.9400±0.0111 |
| **Lung** | 0.9061±0.0285 | 0.8958±0.0346 | 0.9015±0.0280 |
| **Spleen** | 0.9174±0.0263 | 0.9048±0.0266 | 0.9199±0.0218 |
| **Brain** | 0.9550±0.0088 | 0.9467±0.0092 | 0.9121±0.1672 |
| **Thymus** | 0.9065±0.0430 | 0.8881±0.0382 | 0.8621±0.1749 |
| **Heart** | 0.9373±0.0220 | 0.9265±0.0271 | 0.9279±0.0216 |
| **Stomach** | 0.9414±0.0168 | 0.9328±0.0194 | 0.9406±0.0105 |
| **Lt kidney** | 0.9474±0.0306 | 0.9362±0.0247 | 0.8330±0.3202 |
| **Rt kidney** | 0.9338±0.0282 | 0.8988±0.0426 | 0.9245±0.0296 |
| **Bladder** | 0.6419±0.2649 | 0.6470±0.2938 | 0.3223±0.3593 |

Table S4. HD of *2D-slices*, *3D-whole-mouse*, and *3D-patches*, against the manual segmentation of Analyst 1.

|  | **2D-slices** | **3D-whole-mouse** | **3D-patches** |
| --- | --- | --- | --- |
| **Liver** | 1.2436±0.4168 | 1.4846±0.6562 | 1.6478±0.5763 |
| **Lung** | 1.2688±0.5463 | 1.2214±0.7958 | 1.5518±0.8620 |
| **Spleen** | 0.5077±0.2365 | 0.5042±0.1871 | 0.5549±0.2543 |
| **Brain** | 0.5506±0.1452 | 0.7554±0.3155 | 1.0120±1.6306 |
| **Thymus** | 0.3551±0.1159 | 0.4539±0.1786 | 0.6687±0.8962 |
| **Heart** | 0.6952±0.3267 | 0.6927±0.2185 | 0.8111±0.3205 |
| **Stomach** | 0.5912±0.1811 | 0.6400±0.2037 | 0.7757±0.4091 |
| **Lt kidney** | 0.4367±0.1485 | 0.5974±0.3403 | 0.7655±1.2146 |
| **Rt kidney** | 0.5512±0.2248 | 0.6291±0.2430 | 0.5651±0.2694 |
| **Bladder** | 2.1442±8.5638 | 3.4141±8.7102 | 5.3517±6.4454 |
