# Supplementary material for: Deep learning multi-organ segmentation for whole mouse cryo-images including a comparison of 2D and 3D deep networks
Source: Sci Rep. 2022 Sep 7;12:15161. doi: 10.1038/s41598-022-19037-3 (PMC9452525; doi:10.1038/s41598-022-19037-3)
Supplement: Supplementary file 2 — Supplementary Legends. [file 41598_2022_19037_MOESM2_ESM.docx]

**Supplementary movie 1.** Visualization of the color cryo images and corresponding predictions by 2D-slices with 2D contouring and 3D surface rendering. Colors are: liver- red, stomach – green, left kidney - bluish purple, right kidney - dark purple, lungs - light cyan, brain – yellow, thymus – blue, heart - light pink, bladder - dark green, spleen - dark cyan.
